# Research exploring monetary incentives for recruiting and retaining participants in randomised controlled trials: a scoping review

**DOI:** 10.1186/s13063-026-09872-4

**Published:** 2026-06-27

**Authors:** Gloria Mongelli, Joy Adamson, Antonina Yakimova, Camila Piccolo-Lawrance, Adwoa Parker

**Affiliations:** York Trials Unit, Department of Health Sciences, University of York, York, UK

**Keywords:** Trial incentives, Financial compensation, Clinical trial participation, Recruitment, Retention

## Abstract

**Background:**

Poor participant recruitment and retention in randomised controlled trials (RCTs) is a widespread challenge. Offering trial participants monetary incentives (e.g. cash or vouchers) may boost recruitment and retention; however, no reviews have systematically mapped the existing quantitative, qualitative, and mixed-method evidence on this topic. We therefore conducted a scoping review aiming to map the extent and type of available research examining monetary incentives for recruiting and retaining trial participants to identify critical areas where evidence remains sparse.

**Methods:**

We included empirical studies examining the use of monetary incentives for recruiting or retaining participants in RCTs (excluding phase I trials). Case studies, protocols, and reviews without a systematic search strategy were excluded. Searches were run in the ORRCA database until the end of 2019 when it was last updated, and CINAHL, MEDLINE, Embase, and EconLit from 2020 onwards.

**Results:**

Out of 5421 identified records, 145 studies from 141 sources met our inclusion criteria. Monetary incentives were primarily studied in the context of trial retention (39%; 56/145) or trial participation more broadly (33%; 48/145), with many studies not specifying the format (61%; 88/145) or value (40%; 58/145) of monetary incentives in question. Additionally, the vast majority of studies were conducted in the USA (48%; 69/145) or UK (34%; 49/145), with very little research activity in low- and middle-income countries (LMICs) (9%; 13/145). Monetary incentives for recruiting or retaining trial participants were most often explored in the context of cross-sectional surveys (21%; 30/145), of which most involved members of the public being asked about hypothetical trial scenarios (53%; 16/30), rather than individuals actively invited to or involved in a trial.

**Conclusions:**

This scoping review consolidates available research on monetary incentives for recruiting and retaining trial participants. Less research is needed involving members of the public being asked about monetary incentives for a hypothetical trial. Rather, we need more qualitative studies exploring the perspectives of trial staff responsible for their implementation, as well as randomised studies within a trial (SWATs) assessing cost-effectiveness alongside effectiveness. Furthermore, more research is needed exploring the use of monetary incentives in LMICs.

**Supplementary Information:**

The online version contains supplementary material available at https://doi.org/10.1186/s13063-026-09872-4.

## Background

Recruiting and retaining adequate numbers of participants in randomised controlled trials (RCTs) presents a widespread challenge [1], leading to substantial research waste [2]. Providing monetary incentives—defined by the Australian National Health and Medical Research Council [3] as ‘money or in-kind support that is provided to participants to encourage their enrolment or continuing participation in research’—has been proposed as a potential strategy to boost recruitment and retention in trials.

Monetary incentives for improving participant recruitment and retention in trials can take a wide array of forms. They can vary in their format (e.g. cash or vouchers), value (e.g. $2–$500), payment frequency (i.e. once versus several times throughout a trial), structure (e.g. guaranteed vs lottery-style payments), and may target different behaviours (e.g. trial enrolment, completion of follow-up questionnaires, attendance at follow-up visits). Additionally, monetary incentives can be directed at trial participants or at recruiting staff [4]. However, this review will focus on monetary incentives directed at trial participants, which are particularly controversial.

The ethics of paying research participants is widely discussed in the bioethics literature. The main points argued are that payments may undermine autonomous consent when offered at recruitment and lead people to take unreasonable risks [4, 5]. Another concern raised is that payments may primarily incentivise low-income individuals to participate in research, which could lead this disadvantaged group to unfairly bear the risks and burden of research [4, 6].

It is not clear what breadth of research is available regarding monetary incentives for recruiting and retaining trial participants. Whilst previous reviews have explored monetary incentives [7–11], none has utilised a formal scoping methodology to systematically map the extent and nature of quantitative, qualitative, and mixed-method evidence regarding monetary incentives for recruiting and retaining trial participants. Prior work has either: (1) adopted a broad remit including non-monetary and recruiter-level incentives in trials, pay for performance schemes, and health behaviour change interventions without systematically mapping the evidence [11]; or (2) focussed strictly on synthesising quantitative effectiveness from experimental studies [7–10], thereby disregarding qualitative and observational studies. This study addresses this gap by providing a comprehensive map of the existing literature, thereby identifying critical areas where evidence remains sparse.

## Objectives

The objective of this scoping review is to map the extent, range, and nature of quantitative, qualitative, and mixed-method literature examining the use of monetary incentives for recruiting and retaining trial participants in phase II–IV trials. This study design was selected to characterise the landscape of current primary and secondary research in terms of the questions explored, methods used, and outcomes assessed (including ethical issues). By providing a comprehensive overview, the review aims to identify knowledge gaps and methodological inconsistencies that will inform future research.

## Methods

This scoping review was conducted in accordance with the JBI methodology for scoping reviews [12] and is reported in adherence to the PRISMA Extension for Scoping Reviews (PRISMA-ScR) checklist [13] (available in Supplementary File 1). The protocol for this review was registered on the Open Science Framework [14].

### Search strategy

To avoid duplication of effort, we used an existing database for our initial search—the Online Resource for Research in Clinical triAls (ORRCA). ORRCA is a searchable database that consolidates methodological research on recruitment and retention, and is underpinned by extensive systematic searches [15, 16]. Available literature in ORRCA is categorised into different research domains for recruitment and retention (including ‘monetary incentives’); hence, relevant domains were used as search filters (see Supplementary File 2).

Since the ORRCA searches were last run until the end of 2019, we ran additional searches in MEDLINE, CINAHL (via EBSCO), Embase, and EconLit (via Ovid) on November 24, 2024. The individual search strategies for these databases are also given in Supplementary File 2. Reference lists of included studies were hand-searched for additional studies.

### Eligibility criteria

We included studies that reported findings relating to monetary incentives directed at trial participants to encourage them to enrol in or remain involved in a trial. For incentives to be considered monetary, they had to offer a monetary value either directly (such as via cash payments and bank transfers) or indirectly (such as via gift vouchers, lottery-style prize draws, or charitable donations on their behalf). Monetary incentives had to be provided to encourage trial recruitment or retention, rather than being a component of the trial intervention itself or directly reimbursing out-of-pocket costs (e.g. travel or parking costs). Monetary incentives directed at recruiters, sites, and research staff were outside the scope of this review.

A distinction was made between the recipients of the incentives and the participants of the included studies. Whilst this review focussed exclusively on monetary incentives directed at trial participants, the source of evidence could include other stakeholders, provided they were being asked about monetary incentives directed specifically at trial participants. For instance, the perspectives of professional trial stakeholders such as trial recruiters or trial coordinators were eligible where they provided insight into the use of incentives for trial participants.

To render the studies included more relevant to our topic, only studies that specifically mentioned monetary incentives (e.g. compensation, payment for participation, gift vouchers) in their abstract were included, or where an abstract was not available, they had to discuss monetary incentives in the study aims or objectives.

This scoping review specifically examined the use of monetary incentives for participants in (real or hypothetical) randomised controlled trial scenarios. For example, a hypothetical trial scenario could include potential participants being presented with a simulated trial description to assess their stated willingness to join or remain in a study based on different monetary incentive levels, rather than measuring actual behaviour within an active clinical trial. Studies that explicitly assessed monetary incentives for phase I trials, or trials that involved healthy volunteers who did not face any potential direct intervention benefit were excluded. We excluded phase I trials as we were interested in exploring monetary incentives in contexts where trial participants also have the potential to benefit therapeutically or medically from taking part. This clearly differs from phase I trials that primarily involve healthy volunteers exposing themselves to the inherent uncertainties of early-stage testing, and in exchange typically receive considerable monetary compensation that is often the main motivation for enrolling [17, 18]. For this same reason, we also excluded studies looking at monetary incentives for healthy volunteers who did not face any potential direct intervention benefit. Studies that examined phase 1 trials in addition to later-phase trials were included if they presented findings for non-phase 1 trials separately.

Observational case reports that simply described the use of monetary incentives in a host trial without reporting any other relevant outcomes (e.g. preferred incentive design) were excluded. Conference abstracts that presented study findings not published elsewhere were included to minimise publication bias. Only reviews with a systematic search were included, and previous versions of reviews that have since been updated were excluded to present the most current version. Evaluative designs assessing monetary incentives in combination with another recruitment or retention strategy but not in isolation were excluded, as were protocols.

### Study selection

Following the search, all identified citations were imported into the Mendeley Reference Manager (Elsevier, Amsterdam, Netherlands), and duplicates were removed. Sources were then imported into Covidence (Veritas Health Innovation, Melbourne, Australia) for screening. One reviewer (GM) screened all sources initially, with two additional reviewers (AY or CP-L) subsequently screening 25% of studies between them at both the title and abstract, and full text screening stages. Any disagreements that arose between the reviewers during study screening and selection were resolved through discussion, or via input of a third reviewer (AP or JA).

### Data extraction

Data extraction was conducted using a bespoke data extraction template created in Google Sheets (Google, Mountain View, California). Information related to the study reference, study design, source of funding, study setting/context, study population, details of monetary incentives, study outcomes, and the terminology used in the manuscripts to refer to monetary incentives was extracted.

The data extraction sheet was piloted extensively during its development using studies identified in the ORRCA database. Data were extracted by one reviewer (GM), with 50% being checked by a second reviewer (AY or CP-L). Disagreements arising during data checking were resolved through discussion, or with input from an additional reviewer, if required. The data extraction table is available in Supplementary File 3.

### Data analysis

Findings were consolidated using a narrative summary approach, with descriptive statistics being calculated and presented where appropriate. In accordance with JBI methodology for scoping reviews [12], a formal risk of bias assessment was not conducted as the remit of this study was to map the range of strategies and methodological approaches employed, rather than to synthesise individual study findings. Consequently, no assessment of effectiveness or meta-analysis was performed. Evaluation of specific incentive values requires a systematic review, which has been undertaken elsewhere [8, 19]. Instead, results are tabulated by the study design and methods, and whether studies examined monetary incentives for recruitment, retention, or trial participation more broadly. Included studies were categorised into one of six overarching categories: (1) quantitative surveys (e.g. cross-sectional surveys), (2) evaluative studies within a trial (SWATs), (3) evidence syntheses (e.g. systematic reviews), (4) secondary analyses (e.g. secondary analyses of national surveys), (5) primary qualitative designs (e.g. semi-structured interviews), and (6) mixed-method studies (e.g. Delphi surveys). Results are presented according to the overarching study category and/or whether they related to recruitment, retention, or trial participation.

## Results

**Fig. 1:**
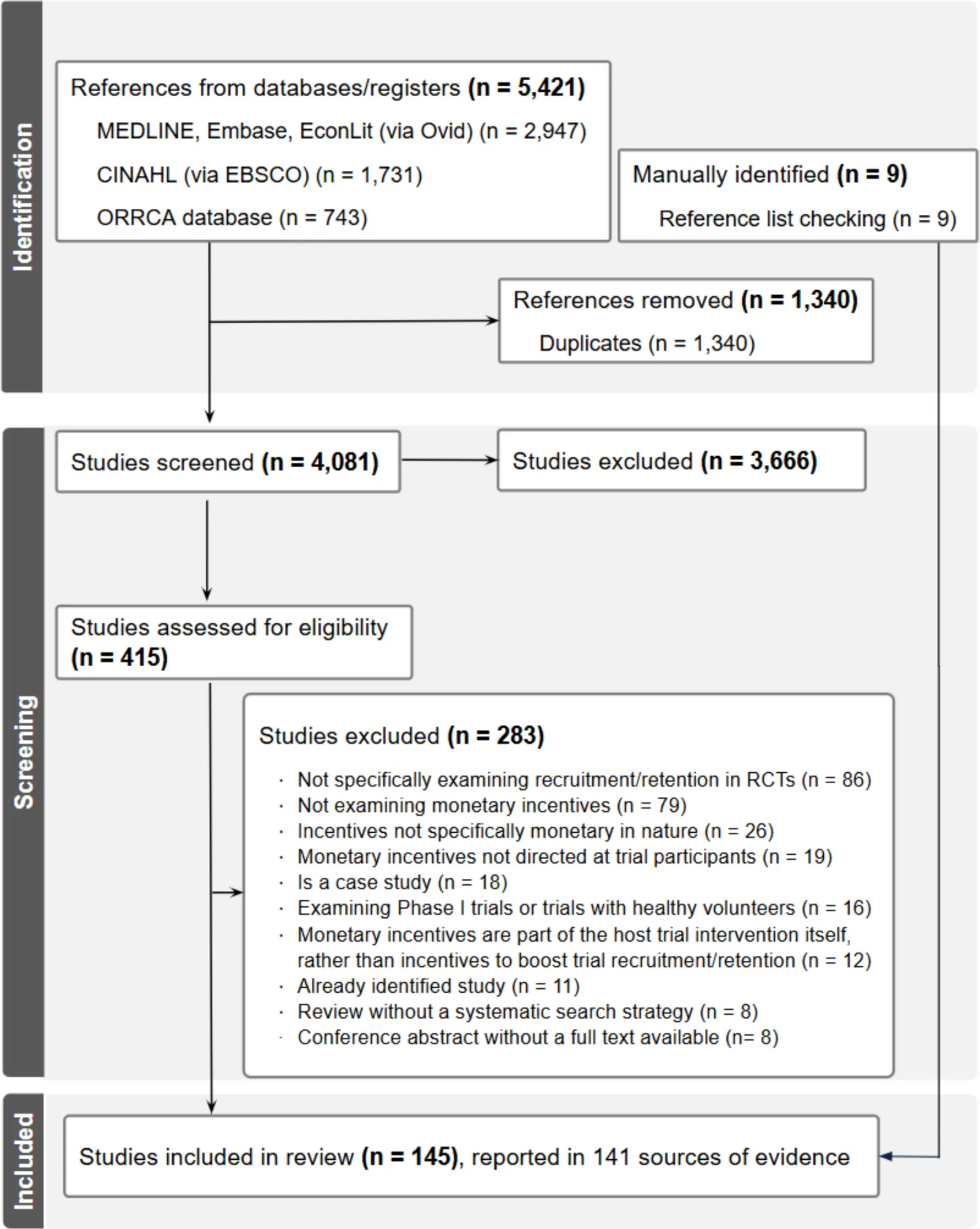
PRISMA flow diagram

We screened 5421 search results, which led to 145 studies from 141 sources of evidence being included in this scoping review (of which 9 were manually identified via reference list checking). Most included sources were academic journal articles (91%; 129/141), with a small number being conference abstracts without a full text available (4%; 6/141) or chapters of a National Institute for Health and Care Research (NIHR) report (4%; 6/141) (Fig. 1).

### Geographical spread

**Fig. 2:**
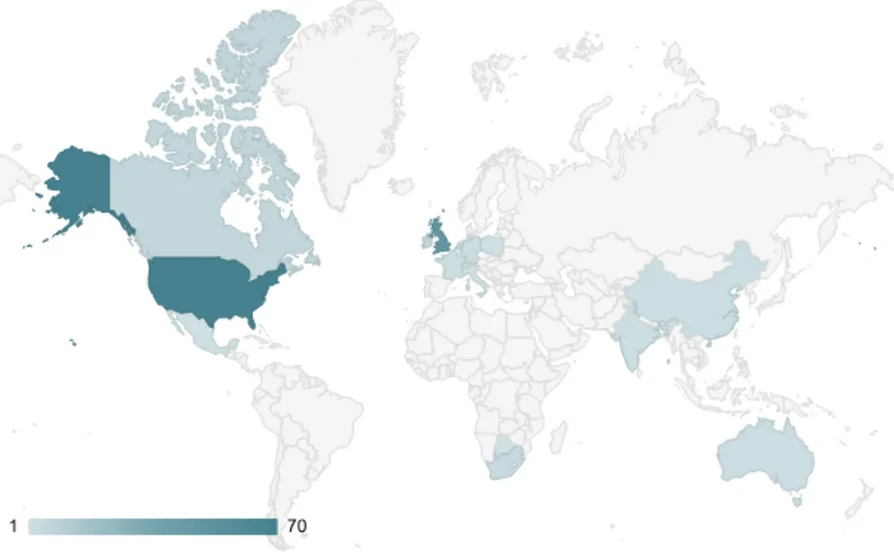
Geographical spread of included studies

Almost all studies were conducted in a single country (94%; 136/145), whilst nine studies were multinational (6%; 9/145). All multinational studies were conducted in either the UK and Republic of Ireland (56%; 5/9) or the USA and one or more other countries (44%; 4/9). Including the multinational studies, the USA contributed almost half (48%; 69/145) and the UK a third (34%; 49/145) of all included studies (Fig. 2). Almost a sixth of studies were conducted in either Europe (excluding the UK) (10%; 14/145), Canada (4%; 6/145), or Australia (2%, 3/145). Only 9% (13/145) of studies took place in low- and middle-income countries (LMICs), namely South Africa, China, India, Mexico, Botswana, and an undisclosed country in West Africa.

### Clinical area

A fifth of studies (20%; 29/145) did not specify a clinical area (Table 1). The most common clinical areas in which monetary incentives were studied were chronic and non-communicable disease (13%; 19/145), sexual health and HIV prevention (10%; 15/145), and smoking cessation (9%; 13/145).

**Table 1:** Characteristics of included studies

Characteristic		*n* = 145
Clinical area	Unspecified	29 (20%)
	Chronic and non-communicable disease	19 (13%)
	Sexual health and HIV prevention	15 (10%)
	Smoking cessation	13 (9%)
	Mental health	11 (8%)
	Other	10 (7%)
	Infectious disease (excluding STIs)	9 (6%)
	Oncology	8 (6%)
	Alcohol and drug abuse (excluding tobacco)	8 (6%)
	Perinatal medicine	6 (4%)
	Paediatrics	6 (4%)
	Mix of primary and specialist care	4 (3%)
	Primary care (general)	3 (2%)
	Gynaecology (excluding perinatal care)	2 (1%)
	Musculoskeletal and orthopaedic medicine	2 (1%)
Source of funding	Public	81 (56%)
	None declared	26 (18%)
	Mix of public & other	12 (8%)
	Unfunded	10 (7%)
	Charity (not-for-profit)	6 (4%)
	Private (academic)	5 (3%)
	Industry (for-profit)	3 (2%)
	Other mix of funders	1 (1%)
	Unclear	1 (1%)
Value of incentives specified	Specify value of monetary incentives	87 (60%)
	Do not specify value of monetary incentives	58 (40%)
Type of incentives*	Unspecified	88 (61%)
	Vouchers*	36 (24%)
	Cash*	13 (9%)
	Bank cheque	4 (3%)
	Reloadable payment card*	4 (3%)
	Lottery-style prize draw*	4 (3%)
	Charity donation*	3 (2%)
	Bank transfer	2 (1%)
Trial scenario	Exploring real-world trial scenarios	106 (73%)
	Exploring hypothetical trial scenarios	32 (22%)
	Exploring both real-world and hypothetical trial scenarios	7 (5%)
Behaviour being incentivised	All retention	56 (39%)
	In-person follow-up	15
	Postal follow-up	11
	Method not specified	11
	Online follow-up	11
	Various follow-up methods available	7
	Telephone follow-up	1
	Trial participation	48 (33%)
	Method not specified	48
	All recruitment	27 (19%)
	Method not specified	14
	Postal recruitment	8
	In-person recruitment	2
	Telephone recruitment	2
	In-person trial registry enrollment	1
	Both recruitment and retention	14 (10%)
	Method not specified	12
	In-person recruitment and retention	1
	In-person recruitment and various follow-up methods	1

*Abbreviations*: *HIV* human immunodeficiency virus, *STIs* sexually transmitted infections

^*^Some studies used more than one type of incentive (e.g. evaluations comparing cash versus vouchers), therefore counts do not add up to *n *= 145

### Source of funding

Almost a fifth of studies (18%; 26/145) did not declare if and how the research was funded. Of the studies that declared funding, the majority were publicly funded (69%; 82/119), 10% (12/119) were funded by a combination of public and other funding, and 8% (10/119) were unfunded (Table 1).

### Recruitment versus retention

Most included studies examined monetary incentives in the context of either trial retention (39%; 56/145) or trial participation (33%; 48/145), the latter not specifying if trial participation related to recruitment, retention, or both (Table 2). Almost a fifth studied monetary incentives for trial recruitment (19%; 27/145), and a tenth examined both recruitment and retention (10%; 14/145).

**Table 2:** Types of included studies by category of study design and incentive context

Research evidence type	Recruitment (*n* = 27)	Retention (*n* = 56)	Both recruitment and retention (*n* = 14)	Trial participation (*n* = 48)	Overall (*n* = 145)
Quantitative surveys	5 (19%)	3 (5%)	0 (0%)	33 (69%)	41 (28%)
Cross-sectional survey not embedded in a trial	1	-	-	15	16
Cross-sectional survey embedded in a trial	2	3	-	9	14
Discrete choice experiment	1	-	-	4	5
Vignette survey	-	-	-	3	3
Randomised hypothetical SWAT	1	-	-	-	1
Stated preference survey	-	-	-	1	1
Expert consultation survey	-	-	-	1	1
Evaluative SWATs	11 (41%)	26 (46%)	0 (0%)	0 (0%)	37 (26%)
Randomised SWAT	7	18	-	-	25
Non-randomised SWAT	4	5	-	-	9
Quasi-randomised SWAT	-	3	-	-	3
Primary qualitative studies	5 (19%)	15 (27%)	7 (50%)	8 (17%)	35 (24%)
Embedded semi-structured interviews	1	6	3	2	12
Non-embedded semi-structured interviews	2	2	3	1	8
Non-embedded focus groups	1	-	1	2	4
Embedded semi-structured interviews and focus groups	-	2	-	1	3
Other mix of qualitative methods	-	3	-	-	3
Qualitative analysis of open-ended survey questions	-	1	-	1	2
Non-embedded semi-structuredinterviews and focus groups	-	-	-	1	1
Embedded focus groups	-	1	-	-	1
Embedded semi-structured interviews and discourse analysis	1	-	-	-	1
Evidence syntheses	4 (15%)	6 (11%)	6 (43%)	1 (2%)	17 (12%)
Systematic review (excluding qualitative)	2	5	5	-	12
Scoping review	-	1	1	1	3
Literature review	1	-	-	-	1
Qualitative evidence synthesis	1	-	-	-	1
Mixed-method studies	1 (4%)	4 (7%)	1 (7%)	2 (4%)	8 (5%)
Multi-phase guidance development project	-	-	1	1	2
Mixed-method survey with open-ended questions	1	-	-	1	2
Delphi survey	-	1	-	-	1
Consensus development workshops	-	1	-	-	1
Co-design workshop	-	1	-	-	1
Qualitative focus groups and quantitative survey	-	1	-	-	1
Secondary analyses	1 (4%)	2 (4%)	0 (0%)	4 (8%)	7 (5%)
Secondary analysis of trial data	1	2	-	1	4
Secondary analysis of a cross-sectional survey	-	-	-	3	3

*Abbreviation*: *SWAT* study within a trial

### Study design category

Overall, over two-thirds of studies were quantitative (70%; 101/145), a quarter were qualitative (25%; 36/145), and 6% (8/145) were mixed-methods. The most common category of study design was quantitative surveys (28%; 41/145), followed by evaluative SWATs embedded in trials (26%; 37/145) and primary qualitative studies (24%; 35/145; Table 2). Evidence syntheses were less common (12%; 17/145), with the fewest being mixed-method studies (6%; 8/145) or secondary analyses (5%; 7/145).

### Study design

Most commonly, studies that explored monetary incentives for recruiting or retaining trial participants were cross-sectional surveys (21%; 30/145), followed by randomised SWATs (17%; 25/145) and semi-structured interviews (14%; 20/145) (see Table 2). Whereas almost half (47%, 14/30) of cross-sectional surveys were embedded in a trial and therefore assessed actual trial experiences, just over half of cross-sectional surveys were not embedded in a trial (53%, 16/30).

### Type of incentives

Almost two-thirds of studies did not specify a type of monetary incentive (61%; 88/145). Of those that did, almost two-thirds studied monetary incentives in the form of vouchers (63%; 36/57), and almost a quarter in the form of cash (23%; 13/57) (Table 1). Additional incentive formats used included bank cheques, reloadable payment cards, lottery-style prize draws, charity donations, and bank transfers (Table 1). Although the majority of studies specified a value of monetary incentives (60%; 87/145), the remaining 40% (58/145) examined monetary incentives without specifying a monetary value (Table 1). The values of monetary incentives offered in real-world trials ranged from $2 to $1000 for participation in a single trial, although the latter was for a 5-year trial that offered between $10 and $75 per trial visit [20]. Hypothetical studies assessed willingness to participate in hypothetical trial scenarios offering up to $2000 [21–23].

### Terminology

**Fig. 3:**
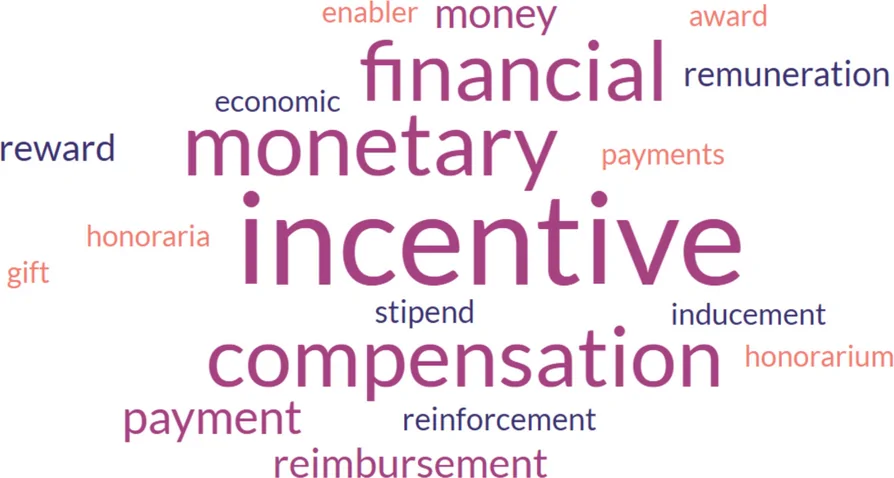
Terminology used to refer to monetary incentives in the included manuscripts

Although a wide range of words were used to refer to monetary incentives in the identified studies (Fig. 3), three quarters of studies (75%; 109/145) used the term ‘incentives’, and 43% (62/145) used the term ‘compensation’, often with the adjectives ‘monetary’ or ‘financial’. Half of all included studies (50%; 73/145) used terms interchangeably, with more than half of these (55%; 40/73) using the terms ‘incentives’ and ‘compensation’ interchangeably. The full list of terminology used is depicted in Fig. 3, although these individual terms were often used in combination (e.g. incentive payment, financial reward, or monetary compensation).

### Study participants and sources of study data

Of the primary study designs (*n* = 121), most included individuals actively invited to or taking part in a trial (62%; 75/121), a quarter involved members of the public not actively being involved in or invited to a trial (25%; 30/121), and a tenth sought the perspectives of professional trial stakeholders such as trial recruiters or trial coordinators (10%; 12/121) (Table 3). Of the evidence syntheses (*n* = 17), many specifically included SWATs (35%; 6/17), which are evaluations of alternative ways of delivering a particular trial process embedded in an RCT [24]. Of the secondary studies (*n* = 7), most analysed real-world trial data (57%; 4/7), and the rest analysed data from (national or international) cross-sectional surveys (43%; 3/7).

**Table 3:** Sources of individual study data by category of study design and incentive context

Source of study data	Recruitment (*n* = 27)	Retention (*n* = 56)	Both recruitment and retention (*n* = 14)	Trial participation (*n* = 48)	Overall (*n* = 145)
Quantitative surveys	5 (19%)	3 (5%)	0 (0%)	33 (69%)	41 (28%)
Members of the public not actively involved in a trial	2	-	-	20	22
Active trial participants	-	3	-	9	12
Professional trial stakeholders (e.g., trial recruiters)	1	-	-	2	3
People being actively invited to a trial	2	-	-	-	2
Mix of professional and lay person stakeholders not actively involved in a trial	-	-	-	1	1
Lay person stakeholders identified via a national research registry	-	-	-	1	1
Evaluative SWATs	11 (41%)	26 (46%)	0 (0%)	0 (0%)	37 (26%)
Trial participants due a trial follow-up	-	24	-	-	24
Participants being actively invited to a trial	10	-	-	-	10
Trial participants who did not respond to an initial follow-up attempt	-	2	-	-	2
Participants being invited to enrol in a trial registry	1	-	-	-	1
Primary qualitative studies	5 (19%)	15 (27%)	7 (50%)	8 (17%)	35 (24%)
Trial participants	1	13	1	2	17
Members of the public not actively involved in a trial	2	-	1	4	7
Professional trial stakeholders	-	1	3	-	4
Individuals who had been invited to a host trial	1	-	1	1	3
Mix of trial participants and trial staff	-	1	1	-	2
Members of target population informing design of a future trial (identified via charities)	1	-	-	-	1
People taking part in an observational study (not a trial) being asked about hypothetical trial participation	-	-	-	1	1
Evidence syntheses	4 (15%)	6 (11%)	6 (43%)	1 (2%)	17 (12%)
Eligible trials	1	1	3	-	5
Eligible SWATs	2	2	1	-	5
Eligible trial methodology studies (not limited to SWATs)	-	1	2	1	4
Eligible non-randomised SWATs	-	1	-	-	1
Eligible trial protocols	-	1	-	-	1
Eligible primary qualitative studies	1	-	-	-	1
Mixed-method studies	1 (4%)	4 (7%)	1 (7%)	2 (4%)	8 (5%)
Mix of professional and trial stakeholders		1	1		2
Professional trial stakeholders	1	1	-	-	2
Trial participants	-	2	-		2
Trial consenters and refusers	-	-	-	1	1
Members of the public not actively involved in a trial	-	-	-	1	1
Secondary analyses	1 (4%)	2 (4%)	0 (0%)	4 (8%)	7 (5%)
Data from a cross-sectional survey	-	-	-	3	3
Trial retention data from a single trial	-	2	-	-	2
Trial participant baseline characteristics and trial outcome data	-	-	-	1	1
Trial screening logs	1	-	-	-	1

*Abbreviation*: *SWAT* study within a trial

### Inclusion of underserved groups

Over a third of included studies (36%; 52/145) specifically involved members of minority or underserved communities (Table 4). The vast majority of these (50%; 26/52) studied age extremes (i.e. children or older adults), followed by ethnic minority groups (23%; 12/52) and lower socioeconomic status groups (15%; 8/52).

**Table 4:** Counts of studies specifically involving a minority or underserved population

Demographic characteristic	Recruitment (*n* = 9)	Retention (*n* = 19)	Both trial recruitment and retention (*n* = 5)	Trial participation (*n* = 19)	Overall (*n* = 52)*
Age extremes	5 (56%)	10 (53%)	4 (80%)	7 (37%)	26 (50%)
Children and adolescents	2	5	3	6	16
Children	1	3	-	-	4
Older adults	2	-	1	1	4
Infants	-	2	-	-	2
Ethnic minority	3 (33%)	2 (11%)	1 (20%)	6 (32%)	12 (23%)
Hispanic	-	1	-	2	3
African American	1	-	-	1	2
African Americans and Latino	1	-	-	1	2
Asian American	1	1	-	-	2
Ethnic minority (unspecified)	-	-	1	1	2
Indigenous Americans	-	-	-	1	1
Lower socioeconomic status individuals	2 (22%)	3 (16%)	-	3 (16%)	8 (15%)
Mental health conditions	1 (11%)	4 (21%)	-	-	5 (10%)
Drug or substance abuse	-	2 (11%)	1 (20%)	2 (11%)	5 (10%)
Victims of physical and/or sexual abuse	-	2 (11%)	-	-	2 (4%)
Multiple comorbidities	1 (11%)	-	1 (20%)	-	2 (4%)
Sexual orientation	-	1 (5%)	-	1 (5%)	2 (4%)
Impaired capacity to consent	-	-	1 (20%)	-	1 (2%)
Location (rural)	-	-	-	1 (5%)	1 (2%)
Veterans	-	-	-	1 (5%)	1 (2%)
Pregnant women	-	-	-	1 (5%)	1 (2%)
Underserved populations (general)	-	-	1 (%)	-	1 (2%)

^*^Some studies included underrepresented groups according to more than one demographic characteristic; therefore, counts do not add up to *n* = 52

### Subgroup analyses

Over a quarter (27%; 29/109) of included quantitative or mixed-method studies performed a subgroup analysis to examine associations between sociodemographic factors (e.g. age, gender) and quantitative outcomes related to monetary incentives (Table 5). Just over a third of these (34%; 10/29) analysed more than one sociodemographic factor, with the maximum being five sociodemographic factors assessed in two studies [25, 26]. The most commonly performed subgroup analyses were related to socioeconomic status (41%; 12/29), followed by age (38%; 11/29) and sex/gender (31%; 9/29).

**Table 5:** Studies that included subgroup analyses of demographic characteristics

Demographic characteristic	Recruitment (*n* = 4)	Retention (*n* = 6)	Trial participation (*n* = 19)	Overall (*n* = 29)*
Socioeconomic status	4 (100%)	3 (50%)	5 (26%)	12 (41%)
Age	1 (25%)	4 (67%)	6 (32%)	11 (38%)
Sex/gender	1 (25%)	3 (50%)	5 (26%)	9 (31%)
Race/ethnicity	-	1 (17%)	5 (26%)	6 (21%)
Level of education	-	1 (17%)	5 (26%)	6 (21%)
Location	-	-	2 (11%)	2 (7%)
Sexuality	-	-	1 (6%)	1 (3%)
Employment status	-	-	1 (6%)	1 (3%)
Veteran status	-	-	1 (6%)	1 (3%)
Trial completers versus non-completers	-	-	1 (6%)	1 (3%)

^*^Some studies performed subgroup analyses for more than one demographic characteristic; therefore, counts do not add up to *n* = 29

### Evaluative designs

The majority of evaluative SWATs were randomised (70%; 26/37), around a quarter were non-randomised (24%; 9/37), and a small portion were quasi-randomised (8%; 3/37). Evaluation of monetary incentives embedded in a trial primarily compared monetary incentives to no incentives (65%; 24/37), followed by comparing the same format different values (e.g. £5 versus £10 vouchers) (24%; 9/37), or comparing conditional versus unconditional incentives of the same value and format (e.g. conditional versus unconditional £10 vouchers) (16%; 6/37).

### Study outcomes

Almost two-thirds of studies were exploring real-world trial scenarios (73%; 106/145), over a fifth were studying hypothetical trial scenarios (22%; 32/145), and a small portion was studying both real-world and hypothetical trial scenarios (5%; 7/145).

Very few included studies examined ethics-related outcomes (i.e. whether decision-making around trial participation was compromised by monetary incentives) (12%; 17/145). For example, one survey assessed whether members of the public would hypothetically be willing to commit fraud by lying about their medical history to participate in a trial that compensated five times a regular compensation [26], whilst another discrete choice experiment that presented respondents with different hypothetical trial scenarios to choose between assessed the influence of payment on risk perception [21].

Of the evaluative SWATs (*n* = 37), almost half did not report cost-effectiveness (49%; 18/37), slightly fewer reported cost-effectiveness (46%; 17/37), and a further two studies reported costs but not cost-effectiveness (5%; 2/37).

## Discussion

The studies included in this scoping review covered a wide range of clinical areas, study designs, populations, and methods of recruitment or follow-up targeted by financial incentives. Most studies examined monetary incentives for trial participants in the context of retention or trial participation more broadly, with many not specifying the value or format of monetary incentives being explored. Monetary incentives for recruiting or retaining trial participants were most often studied via cross-sectional surveys, the majority of which involved members of the public being asked about hypothetical trial scenarios, rather than real-world trial experiences. Additionally, the vast majority of included studies were from the USA or UK, with very little research activity in LMICs.

A fifth of included studies were studying hypothetical trial scenarios, rather than actual trial experiences or practices. It is worth questioning whether hypothetical decisions are consistent with real-world decision-making, particularly as most participants being asked about hypothetical scenarios were members of the public not actively recruited to or retained in a trial. It is for this reason that the recently accepted update of the Cochrane review of strategies to improve recruitment to trials (including monetary incentives) has explicitly excluded hypothetical trials [19]. Not only this, but none of the studies examining hypothetical trial scenarios specified both a value and type of monetary incentive, with most not specifying either. This calls into question the value such studies are contributing to the broader evidence base on this topic, as they are only investigating monetary incentives vaguely.

Furthermore, a large portion of the included studies investigated trial participation more broadly, limiting the usefulness of their results. The term ‘trial participation’ could encompass both trial recruitment and/or retention. That being said, explicitly distinguishing between monetary incentives for trial recruitment and retention rather than solely referring to trial participation may help us understand at what stage of a trial it is most useful to present monetary incentives to prospective or enrolled trial participants to promote sustained trial engagement.

Another important finding was that the terminology surrounding monetary incentives was often used interchangeably and inconsistently both within and between studies. This likely reflects the broad definition of monetary incentives used in this review, where we essentially included any research examining a monetary intervention that was not part of the host trial intervention itself, and did not explicitly involve the reimbursement of direct costs resulting from trial participation (e.g. reimbursement of travel costs). This is because the reimbursement of direct costs is the explicit avoidance of monetary losses, in contrast with the offer of potential monetary gain offered by monetary incentives. The wide variety in terminology used may reflect the various functions served by monetary incentives, such as to compensate participants for their time and effort, thereby acting as an incentive to encourage their continued trial participation. Nevertheless, the term ‘reimbursement’ was still used interchangeably with ‘incentive’ or ‘compensation’ by some included studies, suggesting that monetary incentives may help reimburse any direct costs of participation in the absence of a formal participant reimbursement scheme. Whilst we only captured terminology used in the included manuscripts and conference abstracts themselves, future research could explore how monetary incentives are actually presented to trial participants by exploring what wording is used in participant-facing materials (e.g. participant information sheets).

It is noteworthy that the vast majority (i.e. 81%) of included studies took place in the USA or the UK. This is consistent with the Cochrane reviews of strategies to improve recruitment and retention to trials, where they collectively contributed 69% and 91% of all included studies, respectively [8, 9]. This is unsurprising, given that in recent years the UK has become a major hub for trial methodology research, with initiatives such as Trial Forge and the National Institute for Health and Care Research (NIHR)-Medical Research Council (MRC) Trial Methodology Research Network. Furthermore, in the USA, the inclusion of women and minorities in National Institute of Health (NIH)-funded research became federally mandated under the NIH Revitalization Act of 1993, unless there is a scientifically sound reason to exclude them [27]. This subsequently led to a proliferation of methodological research focussing on improving the recruitment of women and minority groups. Despite the vast amount of research across the USA and UK, there was very little research activity in LMICs, highlighting that more research is needed to examine the use of monetary incentives in these contexts.

### Strengths and limitations

This scoping review was the first of its kind to specifically examine the use of monetary incentives for recruiting and retaining trial participants, and provides a broad overview of the available research addressing this topic. By adhering to the JBI methodology, our review provides a rigorous evidence map that was previously unavailable, identifying specific gaps in the current evidence. Furthermore, by focussing on descriptive and design characteristics, this approach provides necessary context that is frequently lost in traditional systematic reviews of effectiveness.

A limitation of this scoping review is that screening and data checking were not fully completed in duplicate due to resource constraints and the large number of search results identified. Nevertheless, 25% of screening was done in duplicate and 50% of extracted data was second-checked.

A further limitation of this review is that studies were only included if they mentioned monetary incentives in the abstract (or the study objectives in the absence of an abstract), meaning some studies reporting relevant outcomes may have been missed. This was a pragmatic decision following initial piloting of studies identified in the ORRCA database, which showed that otherwise the majority of studies about trial participation would have been brought forward to the full text stage, even though the vast majority did not mention or explore the use of monetary incentives in their results sections. Based on our piloting, this decision would have primarily excluded cross-sectional surveys asking about general motivators for trial participation, where monetary incentives were amongst a broader list of reasons that varied from study to study. This literature is already very saturated, as demonstrated by an umbrella review published in 2020 that examined why patients take part in research [28], which identified a total of 429 unique primary studies amongst 26 systematic reviews—of which 16 reviews were specifically examining participation in trials.

### Implications for research

Our findings reveal a substantial lack of detail in the primary literature, with a significant majority of studies failing to specify the exact type or value of the monetary incentive in question. This poor reporting and/or lack of specificity directly impacts the utility of the existing evidence base. Without a clear description of the incentive’s format, delivery, and context, the evidence remains fragmented, making it difficult to derive meaningful insights into the optimal design, format, and value of monetary incentives for different trial contexts. Future research should explore specific monetary incentive values and incentive types, rather than simply investigating ‘monetary incentives’ as a nebulous, poorly defined concept in cross-sectional surveys, as is often the case.

More randomised evaluations of monetary incentives should assess the cost-effectiveness of monetary incentives, as these may present a considerable cost in trials. For example, a trial with a sample size of 200 participants that presents an unconditional £10 voucher at the primary outcome time point may spend £2000 on incentives alone, and this increases linearly for each additional follow-up time point where monetary incentives are provided. Moreover, there will typically be additional costs involved in administering monetary incentive in trials, such as staff time and postage costs [29]. Nevertheless, the majority of SWATs evaluating monetary incentives did not report their cost-effectiveness. As such, more randomised SWATs assessing cost-effectiveness are needed to help understand whether monetary incentives are a cost-effective use of limited trial resources, and guidance for how to assess cost-effectiveness in a randomised SWAT is now available [30].

Despite the wide debate surrounding the ethics of monetary incentives in the conceptual literature, few of the identified studies examined whether monetary incentives compromise decision-making regarding trial participation, or present other ethical concerns. Such ethics-related considerations are likely best explored using qualitative research designs, where the nuances can be unpicked in more detail than can generally be done in a quantitative study such as a quantitative survey.

Additionally, very few studies investigated the perspectives of professional trial stakeholders, such as trial recruiters, trial managers, or finance representatives. This is a critical stakeholder group, as they are those who actually plan, approve, and deliver monetary incentives to trial participants in the first place. Indeed, we have recently submitted a complementary qualitative evidence synthesis to consolidate the qualitative literature on the use of monetary incentives from the perspectives of relevant stakeholders (including trial participants and trial staff), where we found that insights from trial staff regarding the barriers and facilitators to implementing monetary incentive are lacking in the qualitative literature [31]. Having more qualitative studies specifically involving professional trial stakeholders could give greater insight into potential implementation issues that could inform how monetary incentives are best used in practice.

Whilst further randomised evaluations are needed to determine the ideal structure and format of monetary incentives, investigating the results of the existing demographic subgroup analyses and performing demographic subgroup analyses in any future quantitative studies could give further insight into which demographic groups are most responsive to monetary incentives.

## Conclusions

To conclude, the research examining the use of monetary incentives in trials primarily stems from the USA and the UK, with very little research activity in LMICs. Additionally, many studies do not specify the value or format of monetary incentives in question, and use inconsistent language when discussing them. More quantitative research is needed exploring the cost-effectiveness of monetary incentive in the form of randomised SWATs. Moreover, qualitative research is needed to unpick potential ethical issues surrounding monetary incentive in greater nuance. Likewise, further qualitative research specifically involving trial staff would also give additional insight into potential implementation issues surrounding the use of monetary incentives. Research in this area should focus on real-world (as opposed to hypothetical) trial scenarios.

## Supplementary Information

Supplementary Material 1.

Supplementary Material 2.

Supplementary Material 3.

## Data Availability

All data supporting the findings of this study are available within the paper and its Supplementary Information (Supplementary File 3).
