# Supplementary material for: Research exploring monetary incentives for recruiting and retaining participants in randomised controlled trials: a scoping review
Source: Trials. 2026 Jun 27;27:569. doi: 10.1186/s13063-026-09872-4 (PMC13573473; doi:10.1186/s13063-026-09872-4)
Supplement: Supplementary file 2 [file 13063_2026_9872_MOESM2_ESM.docx]

Appendix 2: Search strategy

**ORRCA Database:**

The ORRCA database was searched from inception for recruitment research relating to ‘F1: Participant monetary incentives’ and retention research relating to ‘B1: Monetary incentives’, respectively, up until the end of December 2019 when their searches were last updated. Recruitment research relating to ‘F3: Non-monetary participant incentives’ and retention research relating to ‘B3: Non-monetary incentives’ were also included, to account for any potential discrepancies in what incentives were classed as monetary incentives (as opposed to non-monetary).

**OVID Database:**

Econlit <1886 to November 14, 2024>

Embase <1974 to 2024 November 19>

Ovid MEDLINE(R) ALL <1946 to November 19, 2024>

1. incentive*.ab,ti,kw.
2. payment*.ab,ti,kw.
3. monetary.ab,ti,kw.
4. money.ab,ti,kw.
5. cash.ab,ti,kw.
6. voucher*.ab,ti,kw.
7. gift card*.ab,ti,kw.
8. inducement.ab,ti,kw.
9. reward*.ab,ti,kw.
10. reinforcement.ab,ti,kw.
11. lotter*.ab,ti,kw.
12. donation.ab,ti,kw.
13. charit*.ab,ti,kw.
14. (financ* adj4 (incentive* or reward* or payment* or compensation or reimbursement or inducement or donation)).ab,ti,kw.
15. 1 or 2 or 3 or 4 or 5 or 6 or 7 or 8 or 9 or 10 or 11 or 12 or 13 or 14
16. recruit*.ab,ti,kw.
17. enrol*.ab,ti,kw.
18. enlist*.ab,ti,kw.
19. accrual*.ab,ti,kw.
20. ((participat$ or recruit$ or enrol$ or enlist or accrual) adj4 trial?).tw.
21. retention.ab,ti,kw.
22. retain*.ab,ti,kw.
23. attrition.ab,ti,kw.
24. follow?up.ab,ti,kw.
25. questionnaire return.ab,ti,kw.
26. ((retention or retain* or attrition or follow?up or questionnaire return) adj4 trial?).tw.
27. 16 or 17 or 18 or 19 or 20 or 21 or 22 or 23 or 24 or 25 or 26
28. RCT*.ab,ti,kw.
29. SWAT.ab,ti,kw.
30. Study within a trial.ab,ti,kw.
31. embedded trial.ab,ti,kw.
32. randomi#ed trial*.ab,ti,kw.
33. randomi#ed controlled trial*.ab,ti,kw.
34. randomi#ed study.ab,ti,kw.
35. clinical trial*.ab,ti,kw.
36. clinical stud*.ab,ti,kw.
37. Clinical Trials as Topic/
38. 28 or 29 or 30 or 31 or 32 or 33 or 34 or 35 or 36 or 37
39. 15 and 27 and 38
40. exp animals/ not humans/
41. 39 not 40
42. 41 not (comment or editorial).pt.
43. limit 42 to yr="2020 -Current"

**EBSCOhost Database:**

CINAHL Ultimate <1886 to 24 November 2024>

| S1 | TI ( incentive* OR payment* OR monetary OR money OR cash OR voucher* OR gift card* OR financ* OR inducement OR reward* OR reinforcement OR economic* OR lotter* OR donation OR charity ) OR AB ( incentive* OR payment* OR monetary OR money OR cash OR voucher* OR gift card* OR financ* OR inducement OR reward* OR reinforcement OR economic* OR lotter* OR donation OR charity ) |
| --- | --- |
| S2 | TI ( recruit* OR enrol* OR retention OR retain* OR attrition OR Follow?up OR accrual* OR participat* OR enlist* OR questionnaire return ) OR AB ( recruit* OR enrol* OR retention OR retain* OR attrition OR Follow?up OR accrual* OR participat* OR enlist* OR questionnaire return ) |
| S3 | TI ( trial* OR RCT* OR SWAT OR randomi#ed trial* OR randomi#ed controlled OR clinical trial* or embedded recruitment trial OR clinical study OR randomi#ed study ) OR AB ( trial* OR RCT* OR SWAT OR randomi#ed trial* OR randomi#ed controlled OR clinical trial* or embedded recruitment trial OR clinical study OR randomi#ed study ) |
| S4 | S1 AND S2 AND S3 |

We subsequently applied a publication start year limit of 2020 onwards via the ‘Edit’ search function.
